# Supplementary material for: RiceChain-Plus: an enhanced framework for blockchain-based rice supply chain systems-ensuring security, privacy, and efficiency
Source: PeerJ Comput Sci. 2025 Jun 11;11:e2926. doi: 10.7717/peerj-cs.2926 (PMC12192870; doi:10.7717/peerj-cs.2926)
Supplement: Supplemental Information 10 [file peerj-cs-11-2926-s010.pdf]

RiceChain-Plus Dataset Documentation

Overview

This dataset accompanies the manuscript titled **"RiceChain-Plus: An Enhanced Framework for Blockchain-Based Rice Supply Chain Systems—Ensuring Security, Privacy, and Efficiency."** It provides a comprehensive collection of data that supports the evaluation and analysis of the RiceChain-Plus framework, a blockchain-based solution tailored for the rice supply chain.

Files Included

- **Device Data**: Information about devices used in the blockchain network.
- **Transaction Cost**: Costs associated with various interactions across tests.
- **Execution Cost**: Costs related to the execution of transactions.
- **Gas Consumption**: Data on gas consumption for transactions.
- **Energy Consumption**: Energy usage metrics in Joules.
- **Execution Time**: Time taken for executing transactions.
- **Transaction Time**: Time taken for processing transactions.
- **Transaction Throughput**: Transaction throughput measurements.

Data Structure Documentation

1. Device Data

- **Description**: Contains cryptographic information related to devices involved in the RiceChain-Plus network.
- **Columns**:
  - **Device Name**: Identifier for each device.
  - **Private Keys**: Cryptographic keys used for secure communication.
  - **Device ID**: Unique identifier for devices in the network.
  - **Message Hash**: Hash value for messages sent by the device.
  - **Signature**: Digital signature for transaction validation.

Example:

Device Name	Private Keys	Device ID	Message Hash	Signature
device_1	0xacf00b...	0xc1ab2e...	405c255...	0x30dffc...

2. Transaction Cost

- **Description**: Provides the cost of transactions across different tests.
- **Columns**:

- **Tests**: Identifier for each test scenario.
- **Intrn-1 to Intrn-100**: Cost metrics for various intrusions and interactions.

Example:

Tests Intrn-1 Intrn-2 ... Intrn-100

test 1 95787 33750 ... 95787

3. Execution Cost

- **Description**: Contains data on the execution cost of transactions.
- **Columns**:
  - **Tests**: Identifier for each test scenario.
  - **Intrn-1 to Intrn-100**: Execution cost metrics for different interactions.

Example:

Tests Intrn-1 Intrn-2 ... Intrn-100

test 1 73331 71294 ... 75607

4. Gas Consumption

- **Description**: Details the gas consumption for each transaction, crucial for blockchain analysis.
- **Columns**:
  - **Tests**: Identifier for each test scenario.
  - **Intrn-1 to Intrn-100**: Gas consumption metrics.

Example:

Tests Intrn-1 Intrn-2 ... Intrn-100

test 1 110156 38813 ... 110156

5. Energy Consumption

- **Description**: Provides data on energy consumption in Joules for each interaction.
- **Columns**:
  - **Interactions**: Identifier for each interaction type.
  - **Avg Execution Cost**: Average execution cost across interactions.
  - **Energy Consumption (Joules)**: Energy usage metrics.

Example:

Interactions	Avg Execution Cost	Energy Consumption (Joules)
Intrn-1	44165.57	0.000093

6. Execution Time

- **Description**: Lists the time taken for executing transactions, measured in milliseconds.
- **Columns**:
  - **Interactions**: Identifier for each interaction type.
  - **Execution Time (ms)**: Execution time in milliseconds.

Example:

Interactions	Execution Time (ms)
Intrn-1	44.165571

7. Transaction Time

- **Description**: Contains data on transaction time in seconds for different interactions.
- **Columns**:
  - **Interactions**: Identifier for each interaction type.
  - **Avg Execution Cost**: Average execution cost across interactions.
  - **Execution Time (ms)**: Execution time in milliseconds.
  - **Transaction Time (s)**: Transaction time in seconds.

Example:

Interactions	Avg Execution Cost	Execution Time (ms)	Transaction Time (s)
Intrn-1	44165.57	44.165571	14.044166

8. Transaction Throughput

- **Description**: Details the transaction throughput measured in transactions per second.
- **Columns**:
  - **Interactions**: Identifier for each interaction type.
  - **Transaction Time (s)**: Transaction time in seconds.
  - **Transaction Throughput (trans/second)**: Throughput rate.

Example:

Interactions	Transaction Time (s)	Transaction Throughput (trans/second)
Intrn-1	14.044166	0.071204

Data Dictionaries

Device Data Dictionary

- **Device Name**: Name of the device.
- **Private Keys**: Cryptographic private keys used by the device.
- **Device ID**: Unique device identifier in the network.
- **Message Hash**: Hash value for messages sent by the device.
- **Signature**: Digital signature for authenticating transactions.

Transaction and Execution Data Dictionary

- **Tests**: Identifiers for each test scenario.
- **Intrn-1 to Intrn-100**: Interaction numbers representing different test scenarios and measurements.
- **Avg Execution Cost**: Average cost of executing transactions across scenarios.
- **Energy Consumption (Joules)**: Energy usage in Joules for specific interactions.

Performance Metrics Data Dictionary

- **Execution Time (ms)**: Time taken for executing transactions, measured in milliseconds.
- **Transaction Time (s)**: Time taken for processing transactions, measured in seconds.
- **Transaction Throughput (trans/second)**: Number of transactions processed per second.
