# Supplementary material for: RiceChain-Plus: an enhanced framework for blockchain-based rice supply chain systems-ensuring security, privacy, and efficiency
Source: PeerJ Comput Sci. 2025 Jun 11;11:e2926. doi: 10.7717/peerj-cs.2926 (PMC12192870; doi:10.7717/peerj-cs.2926)
Supplement: Supplemental Information 11 [file peerj-cs-11-2926-s011.docx]

**Detailed Transaction Flow Explanation**

The RiceChain-Plus framework secures transaction flows using a two-level security model that ensures robust authentication, authorization, and privacy preservation across the rice supply chain.

**First Security Level: Initialization and Mutual Authentication**

The initial security level focuses on validating stakeholders and their devices to prevent unauthorized access and impersonation.

**Step 1: Registration Phase**

- **Input:** Stakeholder details (IDs, roles), Device Identifiers, Ethereum Addresses (EA)
- **Process:**
  1. **Admin** registers all stakeholders’ **Cluster Heads (CHs)** and their corresponding mobile devices.
  2. Each CH generates a **secret key** and uses it to establish a **shared secret** with the Admin.
  3. CH assigns unique identifiers to its sensor nodes.
  4. Stakeholders register their mobile devices by selecting an **ID** and **strong password**, which are hashed and stored securely.
  5. The Admin and CHs store these hashed credentials for future verification.
- **Output:** Registered CHs, mobile devices, and securely stored credentials.

**Step 2: User Device Login Phase**

- **Input:** Stakeholder credentials (ID, password)
- **Process:**
  1. User enters login credentials and selects the CH to interact with.
  2. Mobile devices compute a hashed password and compare it with stored values.
  3. If credentials match, the mobile device proceeds; otherwise, the session is terminated.
  4. A random nonce and timestamp are generated to initiate mutual authentication.
- **Output:** Authenticated user device ready for interaction.

**Step 3: Mutual Authentication Phase**

- **Objective:** Ensure both user and system entities are legitimate before transactions.
- **Process:**
  1. The **Admin Gateway** validates the mobile device using nonces and timestamps to prevent replay attacks.
  2. The **Cluster Head (CH)** verifies the admin’s authenticity using similar cryptographic checks.
  3. Secure session keys are established for encrypted communication.
  4. Mutual authentication is completed when all entities verify each other's legitimacy using cryptographic proofs.
- **Security Measures:**
  1. **Nonces** and **timestamps** prevent replay attacks.
  2. **One-way hash functions** ensure data integrity.
  3. **Mutual authentication** validates both parties.

**Second Security Level: Authorization and Privacy Preservation**

Once mutual authentication is complete, the second security layer manages **access control** and **privacy protection** during stakeholder interactions.

**Step 4: Authorization (RBAC & ABAC)**

- **Objective:** Ensure that only authorized users can access specific data.
- **Process:**
  1. **Role-Based Access Control (RBAC):** Determines data access based on stakeholder roles (e.g., farmer, processor, retailer).
  2. **Attribute-Based Access Control (ABAC):** Enhances RBAC by adding contextual parameters like:
     - **Location-based restrictions**
     - **Time-based access windows**
     - **Data sensitivity levels**
  3. Access requests are evaluated using a combination of roles and attributes before permission is granted.
- **Example:** A processor may access product quality data but cannot view consumer information, while a retailer can view consumer data but not production details.

**Step 5: Privacy Preservation (Zero-Knowledge Proofs - ZKPs)**

- **Objective:** Protect sensitive data while allowing verification.
- **Process:**
  1. **Prover** generates a **Zero-Knowledge Proof (ZKP)** to confirm the validity of sensitive data (e.g., product quality) without revealing the actual values.
  2. The **Verifier** uses proof to confirm authenticity without accessing the raw data.
  3. ZKPs are integrated with blockchain transactions, allowing public verification without data exposure.
- **Example:** A farmer can prove that rice meets quality standards without revealing the specific metrics.
